# Muscle Imaging in Inclusion Body Myositis: Refinement of MRI Criteria and Insights Into Upper Body Involvement

**DOI:** 10.1002/jcsm.70173

**Published:** 2026-01-19

**Authors:** Eleonora Torchia, Matteo Lucchini, José Verdu‐Diaz, Sara Bortolani, Beatrice Ravera, Vincenzo Carlomagno, Alessandra Cicia, Daniela Bernardo, Mauro Monforte, Robert Rehmann, Rudolf Andre Kley, Mario Sabatelli, Jordi Díaz‐Manera, Enzo Ricci, Massimiliano Mirabella, Giorgio Tasca

**Affiliations:** ^1^ Department of Neuroscience Università Cattolica del Sacro Cuore Rome Italy; ^2^ John Walton Muscular Dystrophy Research Centre, NIHR Newcastle Biomedical Research Centre Newcastle University and Newcastle Hospitals NHS Foundation Trust Newcastle upon Tyne UK; ^3^ Unità Operativa Complessa di Neurologia, Fondazione Policlinico Universitario ‘A. Gemelli’ IRCCS Rome Italy; ^4^ NeMO Clinical Center‐Rome, Fondazione Policlinico Universitario A. Gemelli IRCCS Rome Italy; ^5^ Department of Neurology, University Hospital Bergmannsheil Ruhr University Bochum Germany; ^6^ Department of Neurology St. Marien‐Hospital Borken Borken Germany; ^7^ Neuromuscular Diseases Laboratory, Insitut de Recerca de l'Hospital de la Santa Creu i Sant Pau Barcelona Spain; ^8^ Centro de Investigación Biomédica en Red en Enfermedades Raras (CIBERER) Barcelona Spain

**Keywords:** cluster analysis, IBM, inclusion body myositis, muscle MRI, paraspinal muscles, whole‐body imaging

## Abstract

**Background:**

The diagnosis of inclusion body myositis (IBM) can be delayed because of its heterogeneous clinical presentation and the lack of specific biomarkers. Muscle imaging has gained increasing relevance over the past decade and is now included among the supportive criteria in the international diagnostic guidelines. This study aimed to refine MRI criteria for IBM to facilitate clearer pattern recognition, increase their reproducibility and broader clinical applicability. We also aimed to provide a comprehensive evaluation of muscle wasting across the entire body, including less frequently assessed regions such as the neck, scapular girdle and trunk muscles, and to explore the presence of radiological IBM phenotypes through cluster analysis.

**Methods:**

Sixty‐eight MRI scans and clinical records from patients diagnosed with IBM between 2003 and 2024 (60% males; mean age: 66 years, range: 46–85) were retrospectively reviewed. We defined a new set of three main and three supportive MRI criteria based on muscle imaging features and assessed their sensitivity. Whole body muscle involvement was semi‐quantitatively scored using standardized scales across 6006 muscles. Pairwise correlation and K‐means cluster analysis were performed to evaluate clinical–radiological relationships and identify phenotypic subgroups.

**Results:**

The revised MRI criteria achieved 96% sensitivity. Performance was consistent across clinical subgroups and remained robust (83%) in patients with atypical onset. Whole‐body analysis highlighted mild but frequent wasting of paraspinal (90% of scans) and neck and scapular girdle muscles (87%), while intracranial muscles were consistently unaffected. Correlation analysis underlined a significant association between radiological and functional involvement in the lower (*r* = 0.57, *p* < 0.001) but not in the upper body. Cluster analysis revealed two overlapping but distinguishable imaging phenotypes, characterized by different involvement of paraspinal and distal leg muscles. Cluster 1 showed a higher proportion of male patients.

**Conclusions:**

The revised MRI criteria allow a reliable and easy recognition of the IBM pattern of muscle involvement, while whole‐body imaging offers additional insights into disease heterogeneity and supports patient stratification in clinical trials. Clustering results also highlighted a possible sex‐related influence on muscle vulnerability. The observed clinico‐radiological correlations further support the role of muscle MRI indices as a surrogate outcome measure of muscle function.

## 1 Introduction

Inclusion body myositis (IBM) is the most common acquired myopathy in individuals over 40 years of age [1, 2]. Its pathophysiology remains unclear, involving both immune‐mediated and degenerative processes, and no effective treatments are currently available [3]. In the absence of a unique diagnostic biomarker, diagnosis relies on particular myopathological and clinical features [4], which include involvement of quadriceps, flexor digitorum profundus and oropharyngeal muscles leading to dysphagia [5]. IBM prevalence may be underestimated because of slow progression [2] and atypical presentations, which often result in diagnostic delays or misdiagnoses. Despite promising early‐phase results, numerous clinical trials have failed to demonstrate significant benefits [6, 7, 8, 9, 10, 11, 12], further highlighting the urgent need for a more reliable framework to optimize patient selection, stratification and monitoring. As a result, there has been a growing interest in muscle imaging in the workup of suspected IBM patients. Magnetic resonance imaging (MRI) has emerged as a promising tool, offering both diagnostic support and potential in providing outcome measures in clinical trials [13]. Previous research identified an MRI pattern of muscle wasting, mainly based on the peculiar distal thigh appearance [14], which accurately (up to 97%) distinguished IBM from other myopathies. Based on these findings, muscle imaging has recently been incorporated into the available tools to support and expedite IBM diagnosis [13]. Most imaging studies have focused only on lower limbs [15, 16, 17, 18, 19] and forearm muscles [20, 21], while information available on shoulder girdle involvement in muscle MRI assessments is currently limited [15, 22].

Here, we reviewed a cohort of IBM patients and their MRI scans with three main objectives:

1. Refining the MRI criteria for IBM to facilitate easier identification and pattern recognition.
2. Providing a comprehensive MRI assessment of muscle wasting with a focus on neck, scapular girdle and trunk muscles.
3. Identifying possible imaging phenotypes within the IBM spectrum and correlating radiological features with clinical findings.

## 2 Methods

### 2.1 Patient Cohort and MRI Scans

We retrospectively collected data of IBM patients diagnosed between September 2003 and December 2024 and had at least one muscle MRI scan available as a digital file at the neuromuscular disease unit of the Fondazione Policlinico Universitario ‘A. Gemelli’ IRCCS, Rome, Italy, and University Hospital Bergmannsheil, Ruhr University, Bochum, Germany. Patients were classified according to the 2011 ENMC research diagnostic criteria [4]. The following features were gathered from medical records: sex, age, disease duration, walking and swallowing ability, severity and distribution of weakness, and IBM Functional Rating Scale (IBMFRS) scores (including dysphagia, lower limb, upper limb and total scores) at the time of the MRI scan. Additionally, we recorded anti‐cN1A antibody status in the patients' serum [23], when available.

MRI scans of the lower and upper body were acquired using 1.5‐T MRI scanners according to the previously described protocols [14, 24] and visualized using the software ‘Radiant DICOM Viewer’ (https://www.radiantviewer.com). Both T1‐weighted (T1w) and short tau inversion recovery (STIR) sequences were analysed. When available, serial MRI scans performed in the same patient at different time points were included in the analysis, and clinical features/matching of criteria at the time of scan were reported.

### 2.2 Development of the New MRI Criteria

In our previous work, we had defined the lower limb MRI pattern for IBM as characterized by the fatty replacement and atrophy of the distal anterior thigh muscles, predominant over the posterior compartment, alongside with STIR hyperintensity in the same region. These findings constituted the major criteria sufficient to define a typical case and were often associated with involvement of the gastrocnemius medialis and relative sparing of the pelvic muscles, which were considered supporting criteria in case of uncertainty [14].

Here, we have further elaborated these criteria to make the IBM pattern more objectively recognizable and scans easily scorable by non‐experts in muscle imaging, avoiding possible misinterpretations among typical/consistent/atypical judgements and allowing patients to be classified into a binary system (IBM/non–IBM imaging diagnosis). The new MRI criteria were developed by the senior author based on his clinical and imaging expertise and refined collaboratively together with some of the co‐authors (E. R. and M. Mi.), all experienced in neuromuscular imaging and/or IBM. The performance of the newly established criteria was tested both in the ‘old cohort’ (i.e., the one published in our previous work [14]) and in a different retrospective cohort of unpublished scans (i.e., ‘new cohort’). All the available digital MRI scans were examined by two neurologists with neuromuscular expertise (E. T. and M. L).

### 2.3 Whole‐Body Distribution of Muscle Wasting

Muscle involvement through the whole body, with particular focus on the neck, scapular girdle and trunk muscles, was assessed, and its correlations with clinical features were investigated. Five cranial muscles, 13 muscles of the neck and scapular girdle and 34 muscles of the pelvic girdle and lower limbs were evaluated, together with two paraspinal muscle groups at different spinal levels (dorsal and lumbar paraspinal muscles): the transversospinalis (i.e., multifidus) and the erector spinae (i.e., iliocostalis and longissimus).

Consistently with previous works [24, 25], each muscle was individually scored on both sides (with the exception of the tongue) on T1‐weighted images using a four‐point scale (0 = normal; 1 = < 50% fatty replacement; 2 = > 50%; and 3 = complete fatty replacement or atrophy) for the upper body (cranial, neck, scapular girdle, cervical and dorsal trunk muscles) and a five‐point scale (0 = normal; 1 = mild non‐confluent changes; 2 = < 50% fatty replacement; 3 = > 50%; and 4 = complete fatty replacement) for the lower body (pelvic girdle, lower limb and lumbar trunk muscles) [26]. A cumulative upper body T1‐MRI score (UB‐T1 score), a lower body T1‐MRI score (LB‐T1 score) and a total T1‐MRI score (tot‐T1 score, UB‐T1 score + LB‐T1 score) were calculated for each patient as the sum of the individual muscle scores [27].

Asymmetrical involvement was determined by a side‐to‐side score difference in at least one muscle pair, of at least one point in the upper body and at least two points in the lower body [24, 28]. STIR sequences were also evaluated to assess the presence or absence of muscle oedema, defined as signal hyperintensity, in each muscle.

### 2.4 Data Analysis

Descriptive statistics were used to summarize the characteristics of the study cohort. To explore the influence of clinical variables on the performance of the newly proposed criteria, their fulfilment was reported as a whole and across different clinical subgroups. Fisher's exact test was used to assess differences for binary variables, while the chi‐square test was applied to categorical variables, as appropriate. Inter‐rater agreement in evaluating the presence/absence of the new MRI criteria was assessed using Cohen's Kappa (κ). Pairwise correlations were calculated to assess associations between radiological and clinical variables. Normality of continuous variables was evaluated using Fisher's skewness. Pearson's correlation was applied when both variables were normally distributed; otherwise, Spearman's rank correlation was used. Spearman's method was also used for ordinal and numeric–ordinal pairs. Missing values were not imputed because they were randomly distributed and affected only a subset of clinical variables.

T1 scores were re‐scaled to a range between 0 and 100 considering the original scale (four‐point for upper body and five‐point for lower body muscles), and left–right muscle scores were averaged. The processed scores were then standardized using a patient‐wise *z*‐score normalization as in the following equation:

$${\hat{s}}_{m,p}=\frac{s_{m,p}-\mu_{p}}{\sigma_{p}}$$

where $s_{m,p}$ is the intramuscular fat score of the muscle m of patient p, and $\mu_{p}$ and $\sigma_{p}$ are the mean and standard deviation of all scores of patient p, respectively. This highlights deviations from the overall mean involvement and helps in identifying disease stage–independent patterns [29]. Missing data were processed using a mean imputation, and K‐means clustering was used to identify distinct patient subgroups based on their radiological features [30]. The number of clusters was determined by minimizing the silhouette score. Clinical, demographic and serological features were compared across clusters using Kruskal–Wallis for non‐parametric continuous variables, ANOVA for normally distributed ones and chi‐square (χ^2^) for categorical data.

*P*‐values were used to determine statistical significance. Multiple testing was controlled using the Benjamini–Hochberg procedure. A *p*‐value of < 0.05 was considered significant. Statistical analyses and data visualization were performed using Python (Version 3.10). The ethics committee of the Università Cattolica del Sacro Cuore (Rome, Italy; protocol number 5098/14) approved this study. All the scans were acquired for diagnostic or follow‐up purposes. Informed consent for MRI and the use of their data for research was obtained from all participants or their legal representatives.

## 3 Results

### 3.1 Patient Cohort and MRI Scans

A total of 68 MRI scans from 57 IBM patients were analysed. Of these patients, 60% (34/57) were male, and 40% (23/57) were female. Atypical disease manifestations at onset were detected in 20% (11/57) of patients: 7% (4/57) had onset with hyperCKemia, 7% (4/57) with dysphagia and 2% (1/57) each with axial, facial or proximal upper limb weakness.

The ‘old cohort’ consisted of 34 MRI scans, including both the first group of 19 scans and the validation cohort of 15 patients [14]. The ‘new cohort’ comprised 34 additional and unpublished MRI scans.

More than one scan was available for eight patients (*IBM3*, *10*, *15*, *16*, *23*, *35*, *40* and *48*).

At the time of scan, 42 were clinicopathologically defined diagnoses, 15 clinically defined and 11 probable IBM diagnoses. The mean age was 66 years (range = 46–85), and the mean disease duration was 6.7 years (range = 0–18 years). Patients exhibited varying degrees of clinical impairment: 25% (17/68) were able to walk unaided; 46% (31/68) could walk unaided but with difficulty and only for short distances; 28% (19/68) required support to walk; and 1% (1/68) were non‐ambulant. Dysphagia was present in 47% (32/68) of the cases. Anti‐cN1A antibody status was available for 31 patients, and 19 tested positive (Table 1).

**TABLE 1:** Clinical and laboratory features of IBM patients at the time of imaging.

Disease duration	
Mean – years (range)	6.7 (0–18)
≤ 5 years – n. (%)	30 (44%)
6–10 years – n. (%)	18 (27%)
> 10 years – n. (%)	20 (29%)
**Walking ability**	
Ambulant unaided – n. (%)	17 (25%)
Ambulant unaided with difficulties/for short distances – n. (%)	31 (46%)
Ambulant only with support – n. (%)	19 (28%)
Non‐ambulant – n. (%)	1 (1%)
**Swallowing function**	
Dysphagic – n. (%)	32 (47%)
Non‐dysphagic – n. (%)	36 (53%)
**Anti–cN1A antibody status**	
Positive – n. (%)	19 (28%)
Negative – n. (%)	12 (18%)
Not assessed – n. (%)	37 (54%)

### 3.2 Performance of the New MRI Criteria

**FIGURE 1:**
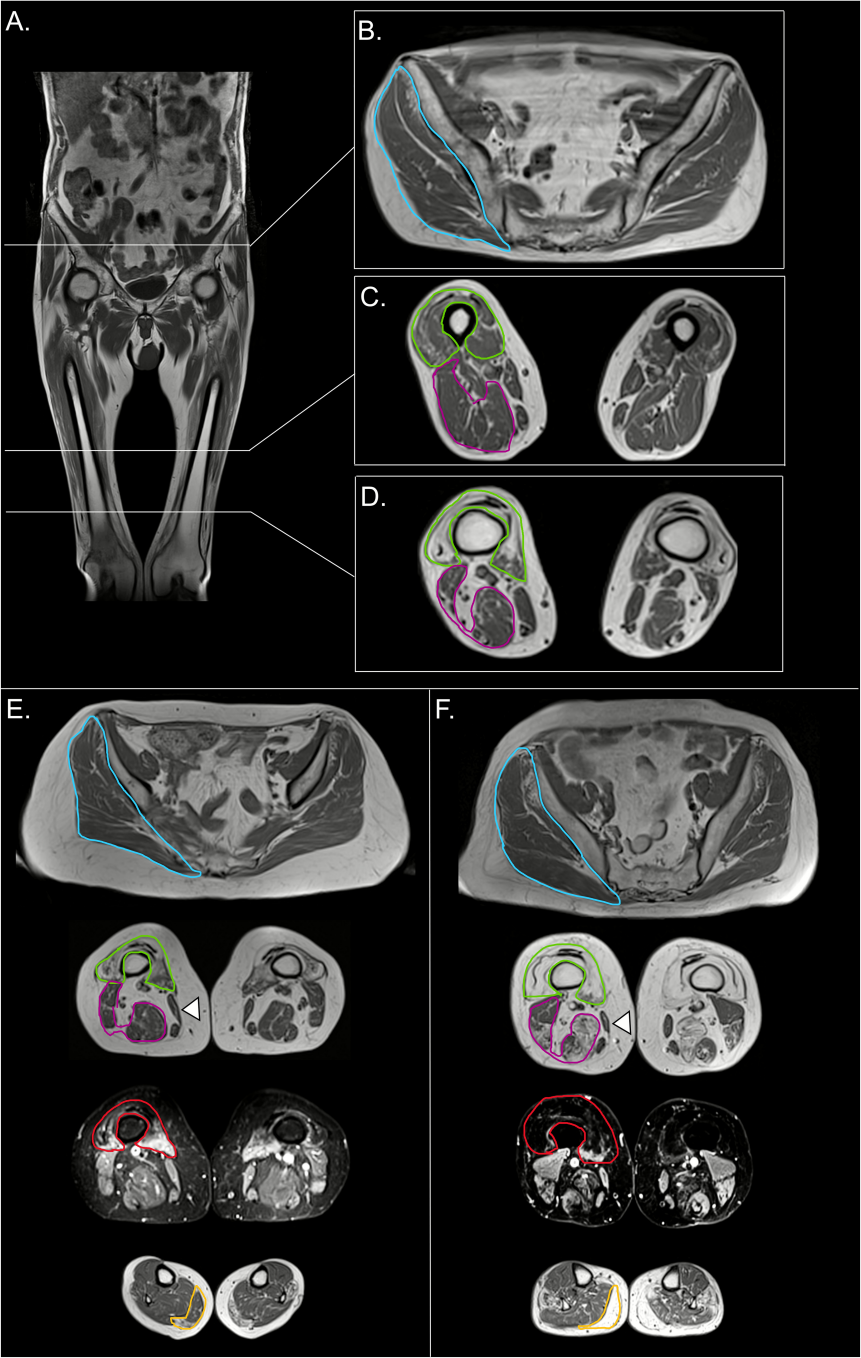
**Refined MRI criteria.** *IBM37* (A–D), *IBM35_1* (E) and *IBM51* (F). (A) T1‐weighted coronal section and corresponding axial slices at pelvis (B) and thigh (C, D) levels. Muscle degeneration of the distal quadriceps (D–F, highlighted in green**, Major Criterion 1**) and hyperintensity on the corresponding STIR image (D–F, outlined in red, **M** **ajor Criterion 2**). At the same level, the anterior thigh is more affected than the posterior compartment (D–F, highlighted in purple, **M** **ajor Criterion 3**). Sartorius muscle (indicated by an arrowhead, **S** **upporting Criterion 1**) often appears hypotrophic and/or shows signs of fatty replacement. Pelvic girdle muscles (e.g., glutei, outlined in blue, and iliopsoas) are relatively spared (**S** **upporting Criterion 2)**. Gastrocnemius medialis (outlined in yellow, **S** **upporting Criterion 3**) shows predominant involvement when the lower leg is affected.

The descriptive refinement of MRI pattern led to the following set of criteria (Figure 1):

#### 3.2.1 Main Criteria

1. Fat replacement of both quadricep muscles in the distal portion (above the knee), particularly involving the vastus intermedius and medialis, or atrophy with a ‘melted’ appearance;
2. Muscle oedema on fat‐suppressed fluid‐sensitive sequences of the distal quadriceps;
3. Predominant involvement of the anterior compartment of the thigh over the posterior one in the distal part.

#### 3.2.2 Supporting Criteria

1. Sartorius involvement;
2. Predominant involvement of the gastrocnemius medialis in the lower leg (at least as affected as the other muscles);
3. Relative sparing of pelvic muscles compared to the thigh.

A diagnosis of IBM requires three main criteria and at least two supporting criteria.

The inter‐rater agreement between the two observers was excellent (κ = 0.87). Discrepancies were reviewed and resolved by consensus. Of the patients, 94% (32/34) of the old cohort and 97% (33/34) of the new cohort could have been diagnosed as IBM at the time of imaging based on the new criteria, leading to an overall sensitivity of 96% (65/68).

The analysis of individual criteria revealed that the main ones showed high consistency: Main Criterion 1 was met by 97% (66/68), while Main Criterion 2 and Main Criterion 3 were each fulfilled in 99% of IBM scans (67/68). Supporting criteria showed more variability: Supporting Criterion 3 was met by all patients, Supporting Criterion 2 by 78% (53/68), and Supporting Criterion 1 by 66% (45/68). A summary of the criteria fulfilment for each scan is provided in Table S1.

The sensitivity of the revised MRI pattern did not differ across clinical subgroups (Table 2) and was not affected by disease duration. When individual criteria were analysed (Table S2), only Main Criterion 1 and Supporting Criterion 1 showed significant associations with clinical variables. Main Criterion 1 sensitivity was slightly lower in atypical than in typical onset (83% vs. 100%, *p* = 0.03), while Supporting Criterion 1 was more frequent in patients with greater walking impairment (*p* = 0.005): 85% in non‐ambulant (17/20), 71% in those walking with difficulties or short distances (22/31) and 35% in those walking unaided (6/17). No other clinical variable significantly influenced the performance of the remaining criteria.

**TABLE 2:** Performance of revised MRI criteria in different clinical subgroups.

	MRI scans fulfilling the revised criteria
**Disease classification** ^a^	**n.s.** ^b^
Clinicopathologically defined IBM – n. (%)	39/42 (93%)
Clinically defined IBM – n. (%)	15/15 (100%)
Probable IBM – n. (%)	11/11(100%)
**Disease onset**	**n.s.**
Typical – n. (%)	55/56 (98%)
Atypical – n. (%)	10/12 (83%)
**Disease duration**	**n.s.**
≤ 5 years – n. (%)	30/30 (100%)
6–10 years – n. (%)	16/18 (89%)
> 10 years – n. (%)	19/20 (95%)
**Swallowing function**	**n.s.**
Non‐dysphagic – n. (%)	35/36 (97%)
Dysphagic – n. (%)	30/32 (94%)
**Walking ability**	**n.s.**
Ambulant unaided – n. (%)	16/17 (94%)
Ambulant unaided with difficulties/for short distances – n. (%)	30/31 (97%)
Ambulant only with support/non‐ambulant – n. (%)	19/20 (95%)
**Anti–cN1A antibody status**	**n.s.**
Negative – n. (%)	12/12 (100%)
Positive – n. (%)	18/19 (95%)

^a^ According to the 2011 ENMC research diagnostic criteria [4].

^b^ **n.s.:** not significant (*p* ≥ 0.05).

Overall, only three scans did not meet the criteria: *IBM6*, *IBM15_3* and *IBM40_1*.

*IBM6* did not fulfil Main Criterion 1, as the quadriceps lacked the characteristic features. The patient, with atypical onset at 54 years, initially presented with axial muscle weakness, gradually progressing to the scapular girdle and severe dysphagia over 7 years. MRI showed predominant fatty replacement in scapular girdle and paraspinal muscles, with relative sparing of lower limbs, except for a subtle hypotrophy of vastus lateralis. Mild knee extensor and finger flexor weakness were clinically evident, and muscle biopsy showed typical IBM features confirming the diagnosis despite the atypical clinical presentation.

*IBM15_3* did not fulfil Main Criterion 2, as no hyperintensity on STIR sequences could be detected in the quadriceps muscles. This scan was performed after 17 years of disease duration and displayed subtotal fatty replacement of the quadriceps, likely reflecting an advanced disease stage without residual inflammation, whereas earlier scans from the same patient (*IBM15_1* and *IBM15_2*, obtained at 13 and 15 years of disease, respectively) met the criteria, still showing STIR hyperintensity in the quadriceps (Figure S1).

*IBM40_1* did not fulfil Main Criterion 1, as there was no significant quadriceps involvement on T1‐weighted images. The patient had a history of asymptomatic hyperCKemia lasting for at least 8 years at the time of this first scan. A follow‐up MRI included in this study (*IBM40_2*, performed 4 years later) instead showed all the typical features (Figure S2).

### 3.3 Whole‐Body Distribution of Muscle Wasting

Out of the 68 MRI scans, 31 comprehended whole‐body MRI studies, derived from the combination of upper and lower body scans from 26 patients (15 males and 11 females), while 37 were scans of the pelvis and lower limbs. Overall, 6006 muscles were scored in the whole cohort.

#### 3.3.1 Cranial, Neck and Scapular Girdle Muscles

Cranial muscles did not show signs of involvement in any of the patients, including the most severely affected ones. Abnormalities in the neck and scapular girdle muscles were noticed in the majority of the scans (87%, 27/31). Pectoralis major was the most frequently affected muscle, involved in 74% (23/31) of cases, followed by latissimus dorsi (65%, 20/31), sternocleidomastoid and subscapularis in 52% (16/31) and serratus anterior in 45% (14/31) of the scans. These abnormalities were mainly characterized by a diffuse hypotrophic appearance and small, non‐confluent areas of abnormal signal (score = 1). Thirty‐nine percent of the scans (12/31) showed asymmetric involvement in at least one muscle. Subscapularis was the most frequently asymmetrically affected muscle (19% of the scans), followed by latissimus dorsi (16%).

Hyperintensity on STIR sequences was noted in 16% (5/31) of the scans, mostly in pectoralis major (13%, 4/31) and latissimus dorsi (10%, 3/31).

**FIGURE 2:**
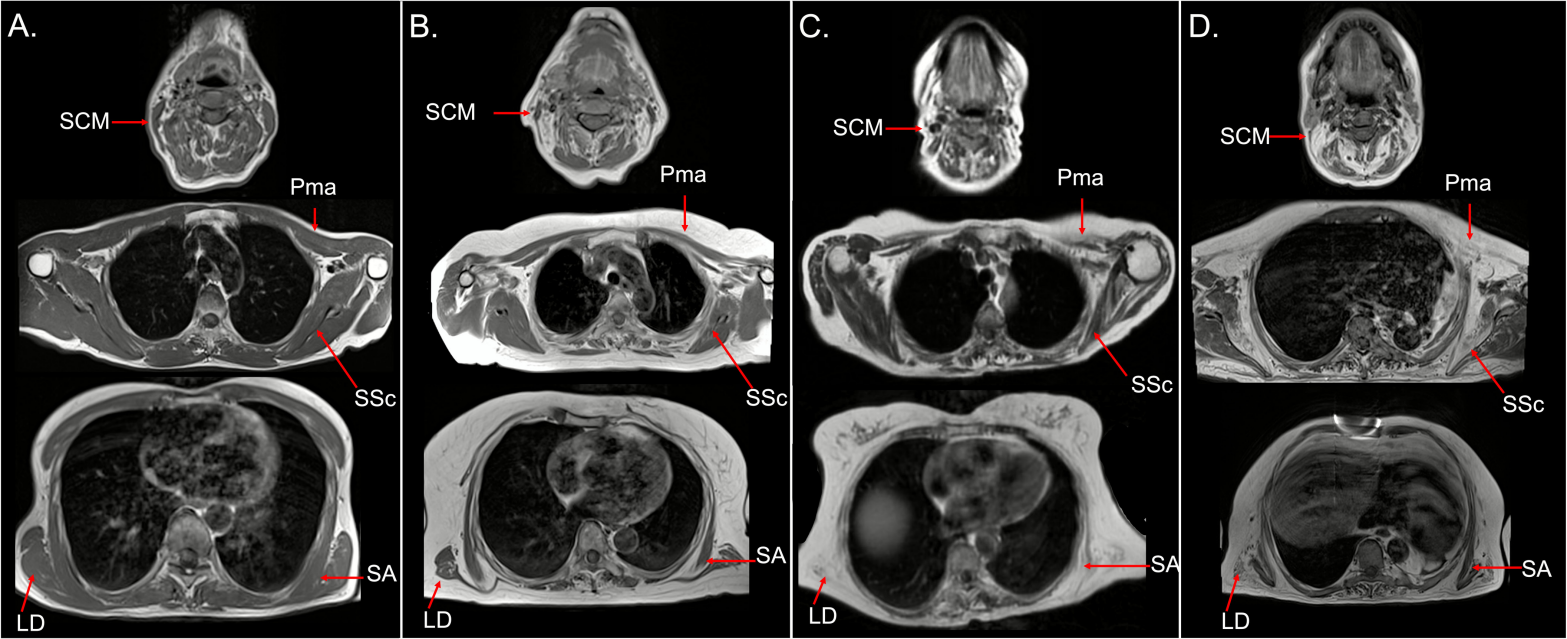
**Neck and scapular girdle involvement in IBM.** T1‐weighted axial images at different levels of the neck and scapular girdle in a 59‐year‐old control (A) and in three patients with different degrees of disease severity (*IBM43* in B; *IBM42* in C; and *IBM19* in D). Patient *IBM43* (B) shows initial signs of scapular girdle involvement (UB‐T1 score = 24) with hypotrophy and mild, non‐confluent fatty replacement, primarily affecting the sternocleidomastoid (SCM), pectoralis major (Pma), latissimus dorsi (LD) and serratus anterior (SA). With increasing severity of upper body involvement (UB‐T1 score = 59 in *IBM42*; UB‐T1 score = 72 in *IBM19*), fatty replacement and atrophy in the same muscles together with subscapularis (Ssc) become more pronounced.

Neck and scapular girdle muscle involvement in IBM is illustrated in Figure 2.

#### 3.3.2 Paraspinal Muscles

**FIGURE 3:**
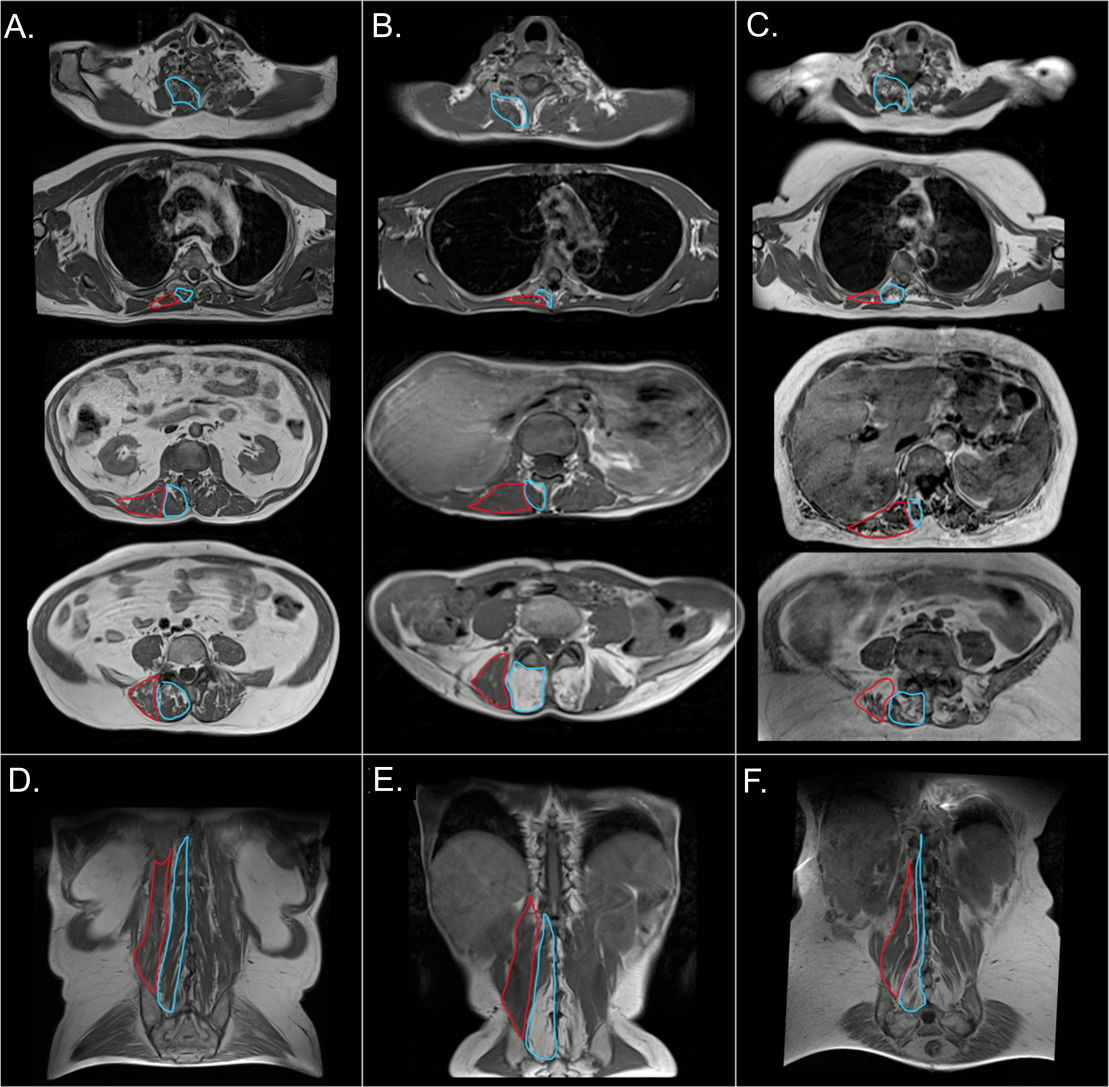
**Paraspinal muscle involvement in IBM.** T1‐weighted MRI scans showing paraspinal muscles in a 59‐year‐old healthy control (A, D) and two IBM patients (*IBM41*, B, E; *IBM50*, C, F) displayed in both axial (A, B, C) and coronal views (D, E, F). Axial slices are at different levels, from cranial (top) to caudal (bottom). Transversospinalis is outlined in blue, while erector spinae muscles are highlighted in red. In *IBM41*, fatty replacement is observed in the transversospinalis muscles, particularly near the rostral and caudal myotendinous junctions. In *IBM50*, a more severe axial involvement is evident, with atrophy of the transversospinalis muscles in the cervical and dorsal regions. In the lumbar region, degeneration is evident in proximity of the sacral insertion.

Paraspinal muscle involvement was detected in 90% (28/31) of the scans. Transversospinalis muscles were the most frequently affected (87% of the scans, 27/31), showing abnormalities in 78% (24/31) of cases at lumbar, in 74% (23/31) at dorsal and in 61% (19/31) at cervical level. This muscle group was mostly hypotrophic and mildly fatty replaced, with a score between 1 and 2 at lumbar level in 68% (21/31) of the scans and a score = 1 at cervical and dorsal levels in 48% (15/31). Erector spinae muscles showed signal abnormalities in 52% (16/31) of cases, mostly caused by mild fatty replacement (mean score = 0.47 at dorsal level and 0.55 at lumbar level). Overall, paraspinal muscle involvement was predominantly observed at the rostral (close to the transverse and spinous processes of the vertebrae) and caudal (posterior sacrum) myotendinous junctions (Figure 3).

Degeneration of abdominal muscles was noticed in 71% (22/31) of the scans, often concomitant with lumbar paraspinal muscle involvement (55%, 17/31).

#### 3.3.3 Lower Body Muscles

Vasti muscles were the most frequently affected, with the vastus lateralis involved in 99% of scans (67/68). A moderate‐to‐severe fatty replacement (score between 2 and 4) was observed in 64% of cases (62/68). The rectus femoris was affected in 56% of scans (38/68), to a lesser extent than the vasti (mean score = 1). The sartorius was also frequently affected although it showed predominantly mild fatty replacement (mean score < 2) in 66% of scans (45/68). Among the medial and posterior thigh muscles, the semimembranosus, gracilis and adductor magnus were the most frequently affected—in 62% (42/68), 56% (38/68) and 50% (34/68) of the scans, respectively—while other muscles were relatively spared. Medial gastrocnemius was the most affected muscle in the lower leg, showing fatty replacement in 82% of scans (56/68), followed by the flexor hallucis longus in 57% (39/68). Both muscles exhibited moderate‐to‐severe involvement (score between 2 and 4) in most cases. The anterior compartment of the lower leg was variably affected, with tibialis anterior and peronei being the most frequently involved muscles, in 53% (36/68) and 49% (33/68) of scans. The pelvic girdle was the least affected region. Gluteus minimus and tensor fasciae latae, which were involved in 63% and 52% of scans, respectively, both showed mild fatty degeneration (mean score < 2). Although asymmetric involvement was observed in at least one muscle pair in 62% of cases (42/68), it was not a major feature: Only 24% of scans (16/68) showed asymmetry in more than one muscle pair. The distribution of asymmetry was variable, with the most frequently asymmetric muscles being medial gastrocnemius (12% of scans, 8/68), tensor fasciae latae (7%) and gluteus minimus (6%). On STIR sequences, each patient exhibited hyperintensity in an average of 14 lower body muscles. Muscle oedema was most frequently observed in vasti (94%), medial gastrocnemius (65%), flexor hallucis longus (47%), tibialis anterior (49%) and adductor magnus (43%).

### 3.4 Cluster and Correlation Analysis

#### 3.4.1 Heatmap

**FIGURE 4:**
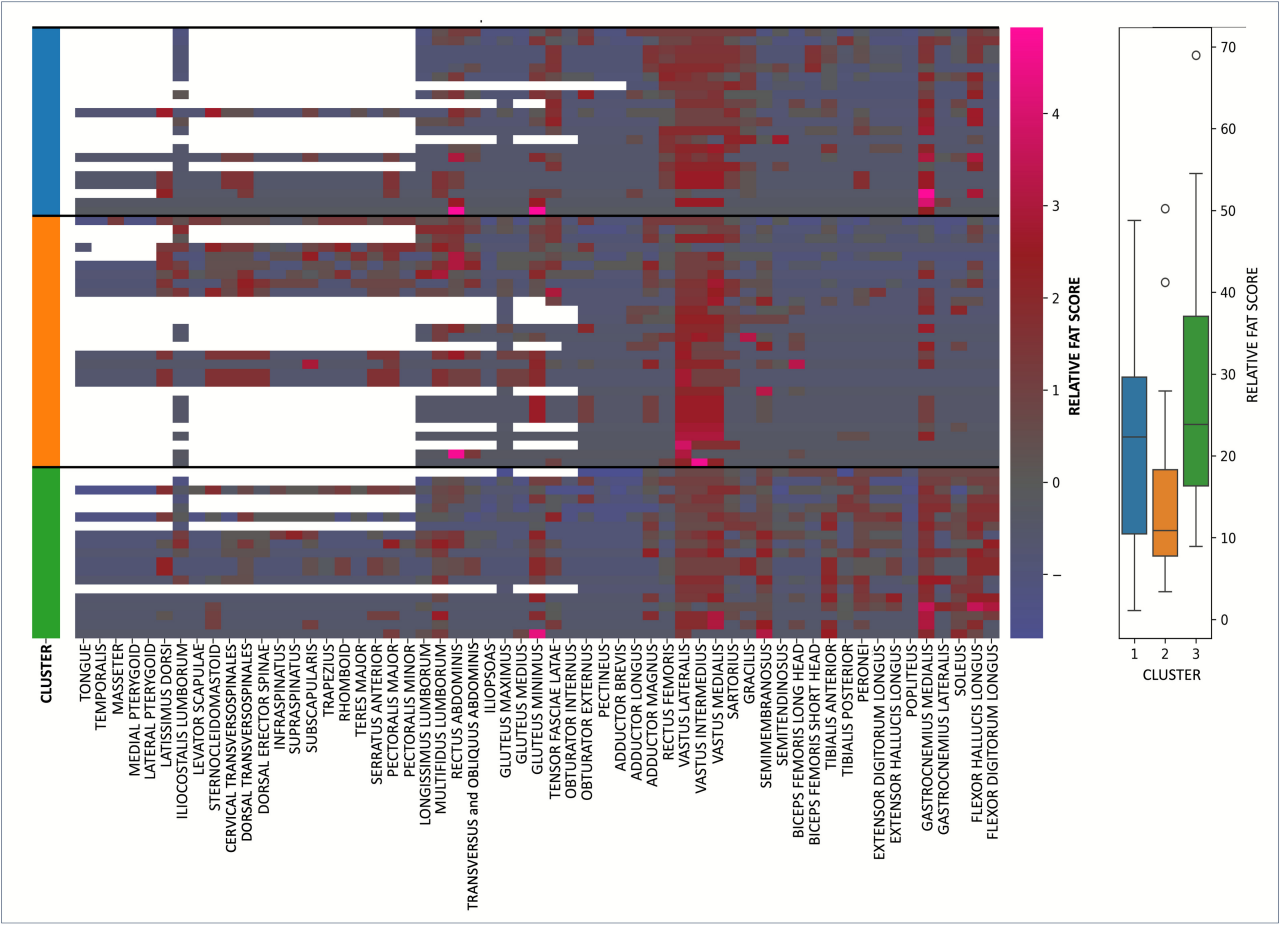
**Unsupervised hierarchical clustering. Muscles are displayed in columns and patients in rows.** Each cell is colour coded according to the *z*‐score derived from the corresponding T1 score, with the gradient reflecting the degree of fatty replacement (see adjacent legend). White cells indicate missing data. The colour‐coded bar on the left denotes patient clustering into three groups: Cluster 1 (blue), Cluster 2 (orange) and Cluster 3 (green). Cluster 2 showed the lowest overall relative fatty replacement, followed by Cluster 1 and then Cluster 3, as illustrated in the boxplot on the right.

The intramuscular fat scores, processed as described in Section 2.4, and the identified MRI clusters are represented as a heatmap in Figure 4. This analysis revealed three clusters, which could be grouped into two partially overlapping radiological phenotypes (Cluster 1 + 3 and Cluster 2). All clusters shared the key MRI features of IBM previously defined through global visual assessment.

– Cluster 1: The semimembranosus muscle was less affected than in other clusters, while the gastrocnemius medialis and flexor hallucis longus muscles were more involved than in Cluster 2. Cluster 1 included a significantly higher proportion of male patients (*p* = 0.02).

– Cluster 2: Paraspinal muscles showed greater involvement, while distal leg muscles, including the gastrocnemius medialis, remained less affected.

– Cluster 3: The gastrocnemius medialis and flexor hallucis longus muscles were as affected as in Cluster 1, with additional involvement of the peroneus, tibialis anterior, extensor hallucis longus and flexor digitorum longus muscles. Cluster 3 was associated with the most pronounced distal weakness in the lower limbs and the greatest impairment in walking ability (both *p* = 0.04).

No other significant differences in clinical or serological features were observed across clusters (Figures S3 and S4).

#### 3.4.2 Correlation With Clinical Findings

Both the LB‐T1 and tot‐T1 scores showed a positive correlation with disease duration (*r* = 0.53 and *r* = 0.46 respectively, *p* < 0.001), lower limb proximal weakness (*r* = 0.44 and *r* = 0.38, *p* < 0.001), lower limb distal weakness (*r* = 0.34 and *r* = 0.36, *p* < 0.01) and walking impairment (*r* = 0.57 and *r* = 0.54, *p* < 0.001). Tot‐T1 and LB‐T1 scores also negatively correlated with the IBMFRS lower limb subscore (*r* = −0.54, *p* < 0.001 and *r* = 0.45, *p* < 0.01, respectively), while no significant correlation was found between the UB‐T1 score and the IBMFRS upper limb subscore. Lower limb proximal weakness and walking impairment were associated with disease duration (*r* = 0.33; *p* = 0.01). Finally, anti‐cN1A antibody status was not significantly associated with any clinical or radiological variable. Detailed correlation analysis results are available in Figure S5.

## 4 Discussion

In this study, we refined the MRI pattern and retrospectively assessed its sensitivity in a multicentre cohort of confirmed IBM patients. Additionally, we expanded the analysis to include imaging data on the upper body, a region less frequently explored, and provided a comprehensive evaluation of whole‐body muscle wasting across various stages of disease severity in IBM.

The refined criteria demonstrated an overall sensitivity of 96%, slightly higher than the 94% reported in our previous study [14]. The main MRI features—fatty replacement or atrophy and oedema in the distal quadriceps, with an anterior predominance of thigh involvement—were highly consistent, with nearly all patients meeting these criteria. Supporting criteria, such as medial gastrocnemius and sartorius involvement, showed greater variability. Notably, sartorius muscle was more frequently involved in patients with walking impairment, suggesting an influence of disease severity on the performance of this criterion.

The MRI criteria performed well also in patients with clinically defined and probable IBM, confirming their reliability even in the absence of all the key clinical or histopathological features. Additionally, these criteria showed high sensitivity in patients with atypical onset, further underscoring the usefulness of imaging in IBM diagnosis, particularly when other diagnostic findings are inconclusive. Their sensitivity did not significantly differ across clinical subgroups—identified based on disease severity, duration and serological features—supporting their broad applicability. However, it is noteworthy that in two of the three cases where the criteria were not met, the patients had another MRI at a different time point that fulfilled them. This suggests that, although disease duration did not significantly influence the overall sensitivity of the revised pattern in our study, the timing of imaging could have an impact on its diagnostic effectiveness in specific cases: patients in the very early stages of IBM may not yet exhibit the characteristic MRI features, making a follow‐up MRI advisable in cases of suspected early IBM. Conversely, in advanced disease stages, widespread fatty replacement may obscure the typical imaging pattern, potentially reducing sensitivity.

Muscle ultrasound represents an alternative imaging method that allows reliable assessment of distal muscles, such as deep finger flexors [31], often not fully captured by usual MRI protocols. However, MRI is essential for evaluating whole‐body imaging patterns, and although ultrasound offers a cost‐effective option, its diagnostic value in distinguishing IBM from other conditions remains untested.

In the second part of this study, we semi‐quantitatively analysed muscle wasting across the neck, scapular girdle, trunk and lower limbs, particularly highlighting the frequent involvement of the paraspinal muscles. Although subsets of IBM patients clinically exhibit axial weakness with camptocormia and dropped head [1, 32, 33], MRI evidence confirming paraspinal muscle wasting has been limited and undetailed [14, 22]. Our findings also revealed a consistent distribution of fatty replacement in the paraspinal muscles, with the transversospinalis being more affected than the erector spinae muscles, predominantly near the myotendinous junction. We acknowledge that age‐related changes and reduced mobility may contribute to these findings. Nonetheless, this consistent presence suggests a possible relevance of axial muscle involvement in IBM. Given the lack of standardized clinical scales for axial muscle assessment, MRI‐based evaluation of paraspinal muscles could provide an objective and quantifiable measure of axial involvement and progression, potentially serving as an outcome measure in clinical trials. This approach may help monitor treatment response and inform therapeutic strategies aimed at preserving axial muscle function.

Scapular girdle imaging did not reveal a distinctive pattern of fatty replacement, with most patients exhibiting mild, non‐confluent changes, particularly in the pectoralis major, latissimus dorsi, sternocleidomastoid and subscapularis muscles. Despite this, upper body imaging may provide added diagnostic value in specific circumstances. Facioscapulohumeral muscular dystrophy (FSHD), for instance, which can mimic IBM with late‐onset facial weakness and inflammatory biopsy findings [34, 35], can occasionally show lower limb imaging features compatible with IBM but shows peculiar findings on upper girdle imaging, including subscapularis sparing [36, 37].

Cranial muscle analysis showed no abnormalities, particularly in the tongue, despite dysphagia being a prominent symptom in many patients. This suggests that tongue weakness is not a primary contributor to dysphagia in IBM, consistent with evidence indicating that upper oesophageal dysfunction and impaired pharyngeal contractions play instead a major role [38, 39].

At the lower body level, semi‐quantitative analysis aligned with previous studies [14, 15, 16, 17, 18, 19], confirming that the quadriceps and medial gastrocnemius are the most severely affected muscles in IBM, with relative sparing of the pelvic girdle and hamstrings. In addition to what was previously described, we also observed a relatively frequent wasting of the flexor hallucis longus.

Cluster analysis revealed the presence of two radiological phenotypes, defined by different degrees and distribution of muscle involvement, all sharing the typical IBM imaging features. Clusters 1 and 3 appeared to represent a continuum of the same phenotype, characterized by a prominent wasting of calf muscles. Cluster 3 showed a further disease progression to distal anterior leg muscles compared to Cluster 1. Cluster 2 emerged as a distinguishable phenotype, marked by predominant trunk involvement, with relative sparing of most distal leg muscles. Interestingly, Cluster 1 was predominantly composed of male patients, suggesting a possible sex‐related susceptibility of muscle involvement associated with this phenotype. To our knowledge, no previous studies have investigated the effect of sex on MRI patterns of muscle involvement in IBM through multivariate analysis, although some observations suggested sex‐related prognostic differences [5].

Correlation analysis demonstrated a significant association between lower body radiological involvement and functional measures, including the IBMFRS lower limb subscore, walking ability and lower limb muscle weakness. These findings, consistent with previous evidence [40], further support the value of intramuscular fat detected on MRI as a valid surrogate biomarker of muscle strength and function, with potential utility as an objective outcome measure in clinical trials. No association was found between the IBMFRS upper limb subscore and upper body muscle involvement as detected on MRI. This may reflect the scale's focus on distal functions, such as object handling or writing, while our protocol mostly investigates the contribution of proximal and axial muscles to disease burden, therefore constituting a complementary evaluation.

Some limitations of this analysis should be acknowledged. Firstly, the retrospective study design and the fact that MRI scans were evaluated unblinded could introduce observer bias. Secondly, the specificity of the new criteria was not determined in this study. However, it is important to note that this study did not aim to differentiate IBM from other conditions, because the specificity of the typical MRI pattern from which these new criteria were derived has been previously validated in a blinded study against a large control group, demonstrating a very high specificity (97%) [14]. The aim was instead to refine the MRI criteria within a confirmed IBM cohort, improving the clarity of MRI‐based muscle assessment in IBM and making the characteristic pattern easier to recognize, even for centres with limited expertise in neuromuscular imaging. Because the typical MRI pattern has been incorporated into the 2024 ENMC criteria for IBM diagnosis [13], facilitating the recognition of the typical imaging features without losing sensitivity can further streamline the diagnostic process and facilitate patient access to specialized care and clinical trials. Future studies, including both IBM and IBM‐mimicking myopathies recruited in a larger, ideally prospective, multicentre cohort, will be necessary to further assess the performance of the refined MRI features and to externally validate them as robust diagnostic criteria for IBM in clinical and research settings.

In conclusion, muscle MRI is an important tool in the IBM diagnostic workup. Lower body imaging remains pivotal, and the newly refined criteria offer an accurate framework facilitating the recognition of the characteristic IBM pattern. While its widespread use is not necessary with the current evidence, complementary upper body imaging can also be valuable in selected patients, supporting differential diagnosis and providing comprehensive markers for scapular girdle and axial wasting, because current clinical scales may not fully capture proximal and trunk muscle deficits.

## Funding

The authors have nothing to report.

## Conflicts of Interest

The authors declare no conflicts of interest.

## Supporting information


**Data S1:** Supporting Information.

**Figure S1:** Sequential muscle MRI assessments in Patient IBM40. In the initial scan (left), vastus lateralis (VL) and vastus medialis (VM) did not show major changes on T1‐weighted sequences but only mild abnormal signal on STIR images. Notably, initial fatty replacement could be observed in the left gastrocnemius medialis (GM) at the time. After 4 years (right), the involvement of the anterior thigh became typical, and further disease progression in the GM could be recognized. Additional signal alterations (low signal intensity on T1‐weighted and hyperintensity on STIR sequences) are observed in the femoral bone, compatible with bone marrow infiltration from T‐cell large granular lymphocytic leukaemia, a condition linked to IBM (red asterisk).

**Figure S2:** Sequential muscle MRI assessments in Patient IBM15. The first scan (left) was performed after 13 years since disease onset, while the second (right) was repeated 4 years later. STIR sequences initially showed mild abnormalities in the vastus medialis (VM) muscle (white arrows), which were no longer detectable in the second scan, in parallel with further advancement of fatty replacement in the vastus lateralis (VL) and VM.

**Figure S3:** Boxplots of discriminant muscles across clusters identified by the unsupervised analysis. Each panel displays muscles with statistically significant differences (*p* < 0.05) across the three clusters. The y‐axis shows Z‐score–normalized T1 values. The x‐axis indicates cluster membership (Clusters 1, 2 and 3). Muscles are grouped anatomically to highlight regional involvement patterns. Based on *p*‐values, the most discriminant muscles across clusters were the semimembranosus, tibialis anterior and flexor hallucis longus.

**Figure S4:** Distribution of clinical features and sex across clusters (*p* < 0.05). Bar plot showing the distribution of lower limb distal weakness across the three clusters (*p* = 0.038). (A) Severity was graded on x‐axis as follows: 0 = normal, 1 = mild, 2 = moderate and 3 = severe. (B) Distribution of mobility scores across clusters (*p* = 0.038). Categories are defined as follows: 0 = ambulant unaided, 1 = ambulant unaided with difficulties or for short distances, 2 = ambulant only with support and 3 = non‐ambulant. (C) Left: distribution of sex across clusters (0 = male, 1 = female). Right: standardized residuals from the chi‐square test assessing the association between sex and cluster membership. Red and blue indicate positive and negative deviations from expected frequencies, respectively.

**Figure S5:** Scatterplot matrix of clinical and radiological variables (p < 0.05). Each dot represents one patient and is colour‐coded by walking ability as shown by the legend on the right (0 = able to walk unaided; 1 = able to walk unaided with difficulties/for short distances; 2 = able to walk only with support; and 3 = non‐ambulant). KDE plots on the diagonal show the distribution of each variable by walking ability group. LB‐T1 score: lower body T1‐MRI score; TOT‐T1 score: total T1‐MRI score (tot‐T1 score, UB‐T1 score + LB‐T1 score); and IBMFRS lower limb score: IBM Functional Rating Scale lower limb score.

**Table S1:** Clinical features and fulfilment of the new MRI criteria in each patient's scan.

**Table S2:** Sensitivity of main and supportive criteria across clinical subgroups of IBM patients.

## Data Availability

Anonymized data not published within this article will be made available upon request from any qualified investigator.
