# Supplementary material for: Muscle Imaging in Inclusion Body Myositis: Refinement of MRI Criteria and Insights Into Upper Body Involvement
Source: J Cachexia Sarcopenia Muscle. 2026 Jan 19;17(1):e70173. doi: 10.1002/jcsm.70173 (PMC12813550; doi:10.1002/jcsm.70173)
Supplement: Supplementary file 1 — Data S1: Supporting Information. [file JCSM-17-e70173-s008.docx]

**Supplementary Material**

**Table S1:** Clinical features and fulfilment of the new MRI criteria in each patient’s scan.

**Table S2:** Sensitivity of main and supportive criteria across clinical subgroups of IBM patients.

**Fig. S1:** Sequential muscle MRI assessments in patient IBM15. The first scan (left) was performed after 13 years since disease onset, while the second (right) was repeated four years later. STIR sequences initially showed mild abnormalities in the vastus medialis (VM) muscle (white arrows), which were no longer detectable in the second scan, in parallel with further advancement of fatty replacement in the vastus lateralis (VL) and VM.

**Fig. S2:** Sequential muscle MRI assessments in patient IBM40. In the initial scan (left), vastus lateralis (VL) and vastus medialis (VM) did not show major changes on T1-weighted sequences but only mild abnormal signal on STIR images. Notably, initial fatty replacement could be observed in the left gastrocnemius medialis (GM) at the time. After four years (right), the involvement of the anterior thigh became typical, and further disease progression in the GM could be recognized., Additional signal alterations (low signal intensity on T1-weighted and hyperintensity on STIR sequences**)** are observed in the femoral bone, compatible with bone marrow infiltration from T-cell large granular lymphocytic leukaemia, a condition linked to IBM (red asterisk).

**Fig. S3: Boxplots of discriminant muscles across clusters identified by the unsupervised analysis.**
Each panel displays muscles with statistically significant differences (p < 0.05) across the three clusters. The y-axis shows Z-score–normalized T1 values. The x-axis indicates cluster membership (Clusters 1, 2, and 3). Muscles are grouped anatomically to highlight regional involvement patterns. Based on p-values, the most discriminant muscles across clusters were the semimembranosus, tibialis anterior, and flexor hallucis longus.

**Fig. S4: Distribution of clinical features and sex across clusters (p < 0.05). Bar plot showing the distribution of lower limb distal weakness across the three clusters (p=0.038). (A) Severity was graded on x-axis as follows: 0 = normal, 1 = mild, 2 = moderate, 3 = severe. (B) Distribution of mobility scores across clusters (p=0.038). Categories are defined as: 0 = ambulant unaided, 1 = ambulant unaided with difficulties or for short distances, 2 = ambulant only with support, 3 = non-ambulant. (C) Left: distribution of sex across clusters (0 = male, 1 = female). Right: standardized residuals from the Chi-square test assessing the association between sex and cluster membership. Red and blue indicate positive and negative deviations from expected frequencies, respectively.**

**Fig. S5: Scatterplot matrix of clinical and radiological variables (p<0.05). Each dot represents one patient and is color-coded by walking ability as shown by the legend on the right (0=able to walk unaided; 1=able to walk unaided with difficulties/ for short distances; 2=able to walk only with support; 3=non ambulant). KDE plots on the diagonal show the distribution of each variable by walking ability group.**

**LB-T1 score:** lower body T1-MRI score**; TOT-T1 score:** total T1-MRI score (tot-T1 score, UB-T1 score + LB-T1 score)**; IBMFRS lower limb score:** IBM Functional Rating Scale lower limb score
