# Supplementary figures and images for: Muscle Imaging in Inclusion Body Myositis: Refinement of MRI Criteria and Insights Into Upper Body Involvement
Source: J Cachexia Sarcopenia Muscle. 2026 Jan 19;17(1):e70173. doi: 10.1002/jcsm.70173 (PMC12813550; doi:10.1002/jcsm.70173)

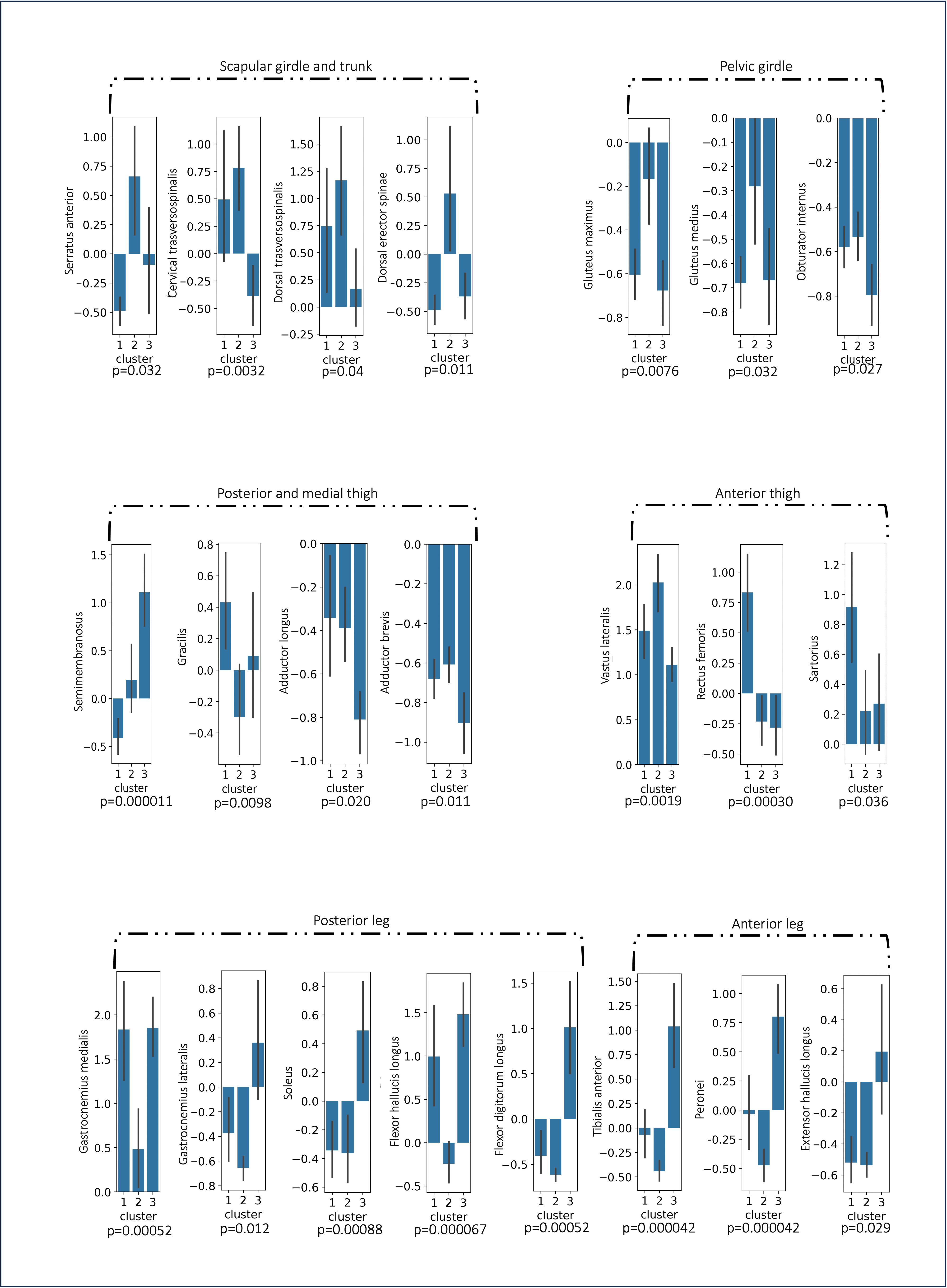

Supplement: Supplementary file 4 — Figure S3: Boxplots of discriminant muscles across clusters identified by the unsupervised analysis. Each panel displays muscles with statistically significant differences (p < 0.05) across the three clusters. The y‐axis shows Z‐score–normalized T1 values. The x‐axis indicates cluster membership (Clusters 1, 2 and 3). Muscles are grouped anatomically to highlight regional involvement patterns. Based on p‐values, the most discriminant muscles across clusters were the semimembranosus, tibialis anterior and flexor hallucis longus. [file JCSM-17-e70173-s006.png]

A.

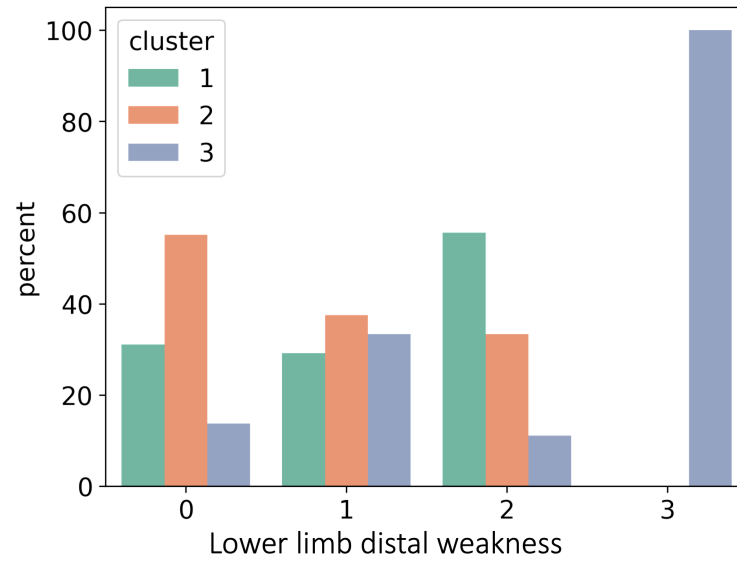

B.

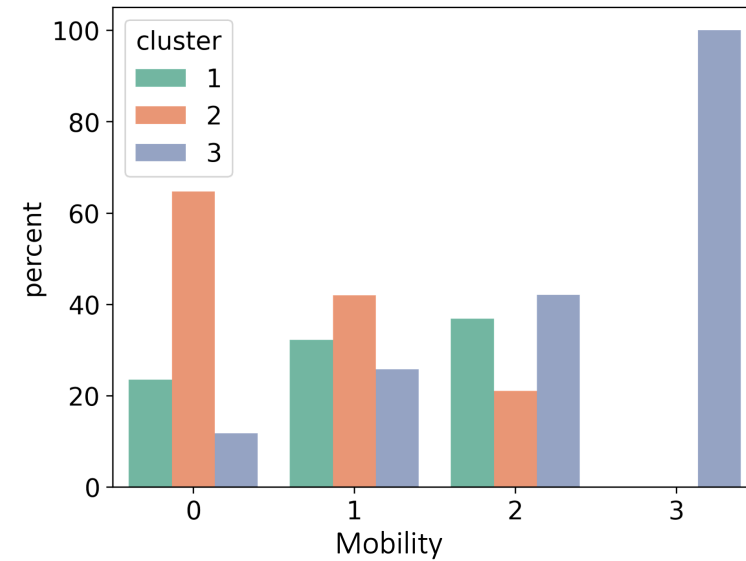

C.

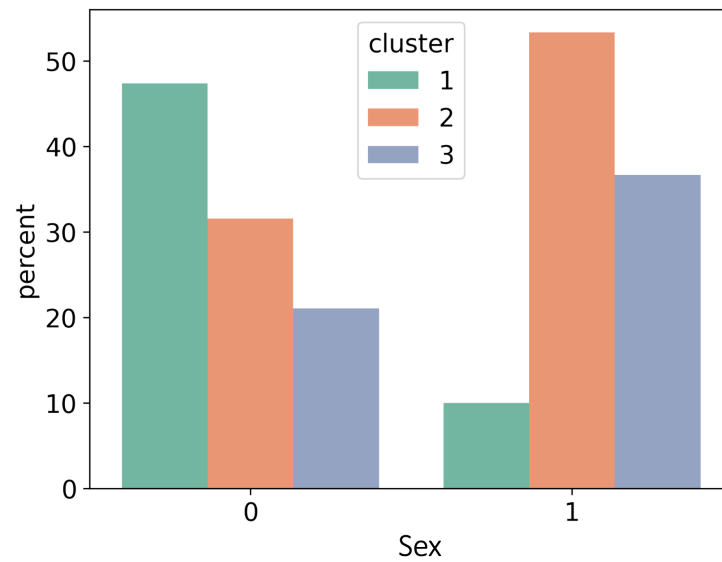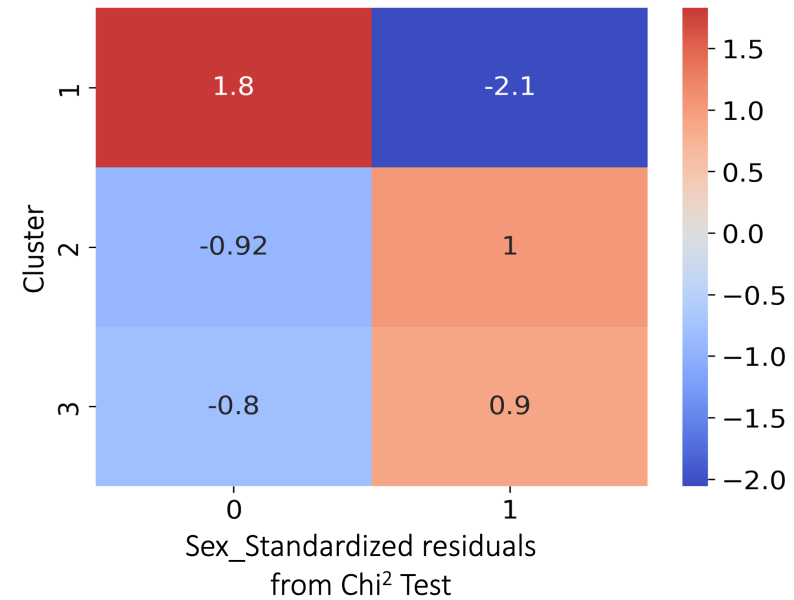

Supplement: Supplementary file 5 — Figure S4: Distribution of clinical features and sex across clusters (p < 0.05). Bar plot showing the distribution of lower limb distal weakness across the three clusters (p = 0.038). (A) Severity was graded on x‐axis as follows: 0 = normal, 1 = mild, 2 = moderate and 3 = severe. (B) Distribution of mobility scores across clusters (p = 0.038). Categories are defined as follows: 0 = ambulant unaided, 1 = ambulant unaided with difficulties or for short distances, 2 = ambulant only with support and 3 = non‐ambulant. (C) Left: distribution of sex across clusters (0 = male, 1 = female). Right: standardized residuals from the chi‐square test assessing the association between sex and cluster membership. Red and blue indicate positive and negative deviations from expected frequencies, respectively. [file JCSM-17-e70173-s004.pdf]

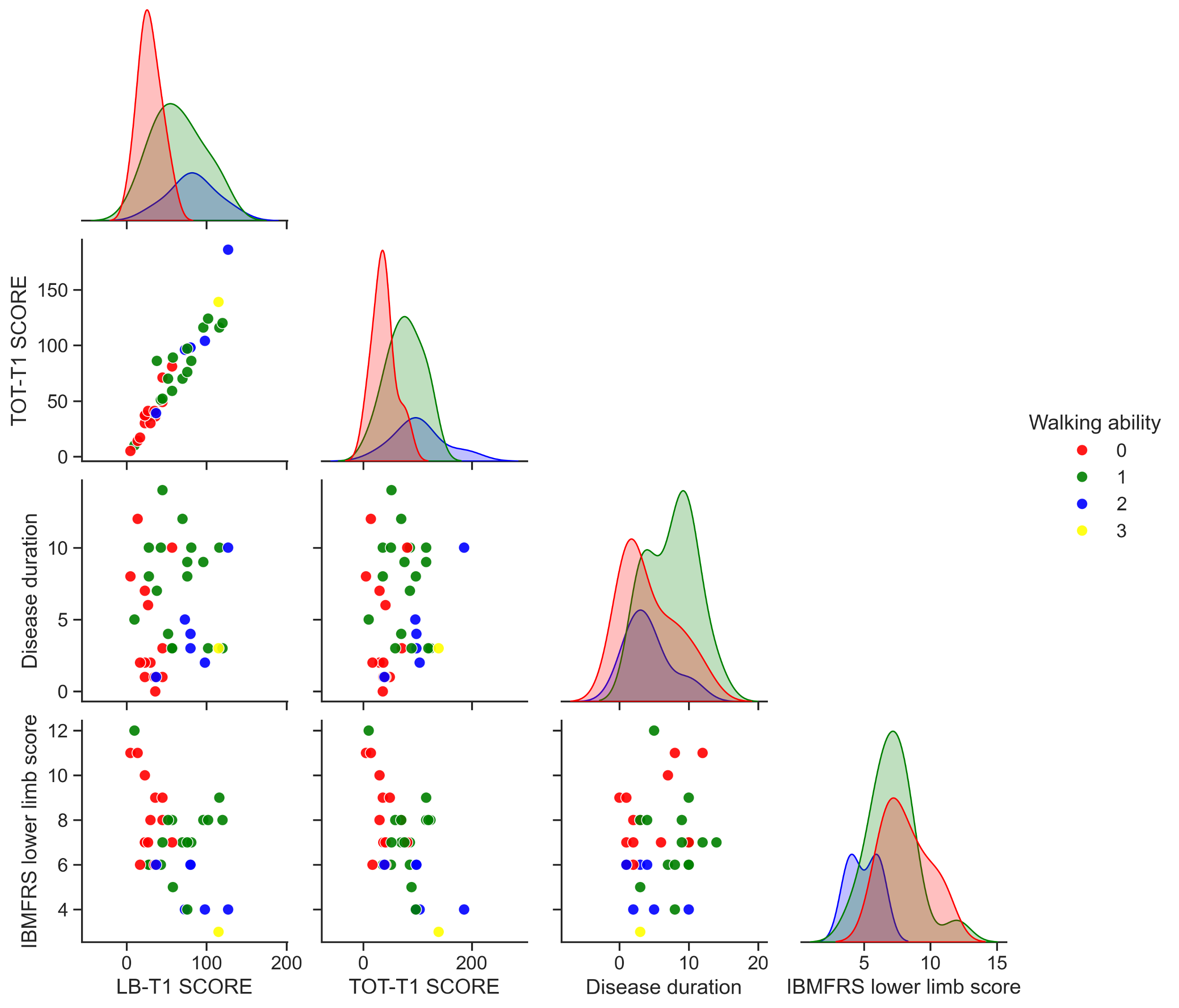

Supplement: Supplementary file 6 — Figure S5: Scatterplot matrix of clinical and radiological variables (p < 0.05). Each dot represents one patient and is colour‐coded by walking ability as shown by the legend on the right (0 = able to walk unaided; 1 = able to walk unaided with difficulties/for short distances; 2 = able to walk only with support; and 3 = non‐ambulant). KDE plots on the diagonal show the distribution of each variable by walking ability group. LB‐T1 score: lower body T1‐MRI score; TOT‐T1 score: total T1‐MRI score (tot‐T1 score, UB‐T1 score + LB‐T1 score); and IBMFRS lower limb score: IBM Functional Rating Scale lower limb score. [file JCSM-17-e70173-s003.png]
