# Supplementary material for: Muscle Imaging in Inclusion Body Myositis: Refinement of MRI Criteria and Insights Into Upper Body Involvement
Source: J Cachexia Sarcopenia Muscle. 2026 Jan 19;17(1):e70173. doi: 10.1002/jcsm.70173 (PMC12813550; doi:10.1002/jcsm.70173)
Supplement: Supplementary file 7 — Table S1: Clinical features and fulfilment of the new MRI criteria in each patient's scan. [file JCSM-17-e70173-s005.pdf]

	Patient number	Scan ID	Sex	Atypical onset	Diagnosis	Age at imaging (years)	Disease duration (years)	Walking ability	Disphagia at MRI	Lower limb proximal muscle weakness at MRI	Lower limb distal muscle weakness at MRI	Upper limb proximal muscle weakness at MRI	Upper limb distal muscle weakness at MRI	IBMFRS total score	IBMFRS dysphagia score	IBMFRS upper limb score	IBMFRS lower limb score	Anti-cN1a antibodies	MAIN CRITERIA M1	M2	M3	SUPPORTING CRITERIA S1	S2	S3	MRI criteria
OLD COHORT	1	IBM1	M	No	Clinico-pathologically defined IBM	73	4	ambulant unaided	Yes	Mild	No	No	Mild					negative	Y	Y	Y	Y	N	Y	Y
	2	IBM2	M	Yes - Dysphagia	Clinico-pathologically defined IBM	85	7	ambulant unaided	Yes	No	Mild	No	Mild	22	0	12	10		Y	Y	Y	N	Y	Y	Y
	3	IBM3_2	M	No	Clinico-pathologically defined IBM	54	1	ambulant unaided	No	Mild	Mild	No	Mild	25	4	12	9		Y	Y	Y	N	Y	Y	Y
	4	IBM4	M	No	Clinico-pathologically defined IBM	57	2	ambulant unaided with difficulties/for short distances	No	Mild	Mild	No	Mild	25	4	12	9		Y	Y	Y	Y	Y	Y	Y
	5	IBM5	M	No	Clinico-pathologically defined IBM	79	10	ambulant unaided with difficulties/for short distances	No	Mild	Mild	No	No						Y	Y	Y	Y	Y	Y	Y
	6	IBM6	F	Yes - Axial weakness	Clinico-pathologically defined IBM	61	7	ambulant unaided with difficulties/for short distances	Yes	Mild	No	Mild	Mild						N	Y	Y	Y	Y	Y	N
	7	IBM7	F	No	Clinico-pathologically defined IBM	80	2	ambulant unaided with difficulties/for short distances	Yes	Mild	No	No	No	19	1	8	6		Y	Y	Y	Y	Y	Y	Y
	8	IBM8	M	No	Clinico-pathologically defined IBM	76	16	ambulant unaided with difficulties/for short distances	No	Moderate	No	No	No						Y	Y	Y	Y	N	Y	Y
	9	IBM9	M	No	Clinico-pathologically defined IBM	78	8	ambulant unaided with difficulties/for short distances	No	Moderate	Mild	No	No						Y	Y	Y	Y	Y	Y	Y
	10	IBM10_1	F	No	Clinico-pathologically defined IBM	65	3	ambulant unaided with difficulties/for short distances	No	No	Severe	No	Moderate						Y	Y	Y	N	Y	Y	Y
	11	IBM11	M	No	Clinico-pathologically defined IBM	58	9	ambulant unaided with difficulties/for short distances	No	Moderate	No	No	No					positive	Y	Y	Y	Y	N	Y	Y
	12	IBM12	M	No	Clinico-pathologically defined IBM	71	14	ambulant only with support	No	Moderate	No	No	No					positive	Y	Y	Y	Y	Y	Y	Y
	13	IBM13	M	No	Clinico-pathologically defined IBM	46	4	ambulant only with support	No	Moderate	No	No	No					positive	Y	Y	Y	Y	Y	Y	Y
	14	IBM14	F	No	Clinico-pathologically defined IBM	62	6	ambulant only with support	Yes	Severe	Moderate	No	Mild						Y	Y	Y	Y	N	Y	Y
	15	IBM15_3	F	No	Clinico-pathologically defined IBM	65	17	ambulant only with support	Yes	Moderate	Moderate	No	No						Y	N	Y	Y	Y	Y	N
	16	IBM16_2	M	No	Clinico-pathologically defined IBM	61	18	ambulant only with support	No	Moderate	No	No	Moderate						Y	Y	Y	Y	Y	Y	Y
	17	IBM17	M	No	Clinico-pathologically defined IBM	71	9	ambulant only with support	No	Moderate	No	No	Moderate						Y	Y	Y	Y	Y	Y	Y
	18	IBM18	M	Yes - HyperCKemia	Clinico-pathologically defined IBM	61	3	ambulant only with support	Yes	Severe	No	No	Mild						Y	Y	Y	N	Y	Y	Y
	19	IBM19	M	No	Clinically defined IBM	51	10	ambulant only with support	Yes	Severe	No	No	Mild						Y	Y	Y	Y	N	Y	Y
	20	IBM20	F	No	Clinico-pathologically defined IBM	50	5	ambulant unaided with difficulties/for short distances	Yes	Severe	No	No	Mild						Y	Y	Y	N	Y	Y	Y
	21	IBM21	F	Yes - Dysphagia	Probable IBM	82	9	ambulant unaided with difficulties/for short distances	No	Severe	Moderate	No	Moderate					negative	Y	Y	Y	Y	Y	Y	Y
	22	IBM22	F	Yes - Facial weakness	Probable IBM	57	12	ambulant unaided with difficulties/for short distances	No	Severe	Moderate	No	Moderate					negative	Y	Y	Y	N	Y	Y	Y
	23	IBM23_1	M	No	Clinico-pathologically defined IBM	72	4	ambulant only with support	No	Severe	Moderate	No	Moderate						Y	Y	Y	Y	N	Y	Y
	24	IBM24	M	No	Clinically defined IBM	69	3	ambulant unaided with difficulties/for short distances	Yes	Mild	Mild	Mild	No						Y	Y	Y	N	Y	Y	Y
	25	IBM25	M	No	Clinico-pathologically defined IBM	80	11	ambulant unaided with difficulties/for short distances	Yes	Moderate	Moderate	Mild	Moderate						Y	Y	Y	Y	Y	Y	Y
	26	IBM26	M	No	Clinico-pathologically defined IBM	72	2	ambulant unaided with difficulties/for short distances	Yes	Mild	No	No	Mild	34	3	15	12		Y	Y	Y	N	Y	Y	Y
	27	IBM27	M	No	Clinico-pathologically defined IBM	69	1	ambulant unaided	Yes	Mild	Mild	No	No	28	0	18	8		Y	Y	Y	N	Y	Y	Y
	28	IBM28	F	No	Clinico-pathologically defined IBM	64	6	ambulant unaided with difficulties/for short distances	Yes	Moderate	Moderate	No	No	34	3	18	7	positive	Y	Y	Y	Y	Y	Y	Y
	29	IBM29	M	No	Clinico-pathologically defined IBM	58	2	ambulant unaided with difficulties/for short distances	No	Mild	Mild	No	Mild	24	4	10	6	positive	Y	Y	Y	Y	N	Y	Y
	30	IBM30	F	No	Clinico-pathologically defined IBM	72	13	ambulant only with support	No	Mild	Mild	No	Mild	24	4	10	6	positive	Y	Y	Y	Y	N	Y	Y
	31	IBM31	M	No	Clinico-pathologically defined IBM	76	7	ambulant unaided with difficulties/for short distances	No	Moderate	Mild	No	Mild	28	4	12	8		Y	Y	Y	Y	Y	Y	Y
	32	IBM32	F	No	Clinico-pathologically defined IBM	61	6	ambulant unaided	No	Severe	mild	No	Mild						Y	Y	Y	Y	Y	Y	Y
	33	IBM33	M	No	Clinico-pathologically defined IBM	77	3	ambulant only with support	No	Mild	Mild	No	Mild						Y	Y	Y	Y	Y	Y	Y
	34	IBM34	F	No	Clinico-pathologically defined IBM	51	3	ambulant unaided	No	Mild	Mild	No	No						Y	Y	Y	N	Y	Y	Y
NEW COHORT	3	IBM3_1	M	No	Clinico-pathologically defined IBM	53	0	ambulant unaided	No	Severe	Mild	No	No						Y	Y	Y	N	Y	Y	Y
	10	IBM10_2	F	No	Clinico-pathologically defined IBM	66	4	ambulant unaided with difficulties/for short distances	No	Severe	Mild	Mild	Mild						Y	Y	Y	N	Y	Y	Y
	10	IBM10_3	F	No	Clinico-pathologically defined IBM	67	5	ambulant unaided with difficulties/for short distances	Yes	Severe	Severe	Mild	Severe						Y	Y	Y	N	Y	Y	Y
	15	IBM15_1	F	No	Clinico-pathologically defined IBM	61	13	ambulant only with support	No	Mild	No	No	No						Y	Y	Y	Y	Y	Y	Y
	15	IBM15_2	F	No	Clinico-pathologically defined IBM	63	15	ambulant only with support	No	Mild	No	No	No						Y	Y	Y	Y	Y	Y	Y
	16	IBM16_1	M	No	Clinico-pathologically defined IBM	60	17	ambulant only with support	No	Severe	No	No	No						Y	Y	Y	Y	Y	Y	Y
	23	IBM23_2	M	No	Clinico-pathologically defined IBM	71	3	ambulant only with support	No	Mild	No	Mild	Mild						Y	Y	Y	Y	N	Y	Y
	35	IBM35_1	F	No	Clinically defined IBM	62	8	ambulant unaided with difficulties/for short distances	No	Moderate	No	No	Mild	21	4	11	6	negative	Y	Y	Y	Y	Y	Y	Y
	35	IBM35_2	F	No	Clinically defined IBM	64	10	ambulant unaided with difficulties/for short distances	No	Moderate	No	No	Mild	21	4	11	6	negative	Y	Y	Y	Y	Y	Y	Y
	36	IBM36	F	Yes - hyperCKemia	Probable IBM	76	10	ambulant unaided with difficulties/for short distances	Yes	Severe	No	Moderate	Moderate	22	3	10	9		Y	Y	Y	Y	N	Y	Y
	37	IBM37	M	No	Clinically defined IBM	69	10	ambulant unaided with difficulties/for short distances	Yes	Severe	Mild	Mild	Moderate	18	3	9	6	positive	Y	Y	Y	N	Y	Y	Y
	38	IBM38	M	No	Probable IBM	75	2	ambulant unaided	No	Mild	No	No	No	31	4	19	8	negative	Y	Y	Y	N	Y	Y	Y
	39	IBM39	F	No	Clinico-pathologically defined IBM	72	3	ambulant unaided with difficulties/for short distances	No	Moderate	Severe	No	No	31	4	19	8		Y	Y	Y	Y	Y	Y	Y
	40	IBM40_1	M	Yes - hyperCKemia	Clinico-pathologically defined IBM	54	8	ambulant unaided	No	No	No	No	Mild	31	4	16	11	positive	N	Y	Y	N	Y	Y	N
	40	IBM40_2	M	Yes - hyperCKemia	Clinico-pathologically defined IBM	58	12	ambulant unaided	No	No	No	No	Mild	31	4	16	11	positive	Y	Y	Y	N	Y	Y	Y
	41	IBM41	F	Yes - Dysphagia	Clinically defined IBM	55	10	ambulant unaided with difficulties/for short distances	Yes	Mild	Mild	Mild	Mild	19	0	12	7	negative	Y	Y	Y	N	Y	Y	Y
	42	IBM42	F	No	Clinico-pathologically defined IBM	72	10	ambulant only with support	Yes	Moderate	Mild	Moderate	Mild	15	2	9	4	positive	Y	Y	Y	Y	Y	Y	Y

43	IBM43	F	No	Clinically defined IBM	67	10	ambulant unaided	Yes	Mild	No	Mild	Mild	21	3	11	7	positive	Y	Y	Y	N	Y	Y	Y
44	IBM44	M	No	Clinically defined IBM	64	3	ambulant unaided	Yes	Mild	Mild	No	Mild	24	3	13	8	negative	Y	Y	Y	Y	N	Y	Y
45	IBM45	M	Yes - Dysphagia	Clinico-pathologically defined IBM	66	3	ambulant unaided with difficulties/for short distances	Yes	Mild	Moderate	Mild	Moderate	16	1	10	5	positive	Y	Y	Y	Y	N	Y	Y
46	IBM46	M	No	Probable IBM	70	3	ambulant unaided with difficulties/for short distances	Yes	Moderate	Severe	Mild	Moderate	20	3	9	8	positive	Y	Y	Y	Y	Y	Y	Y
47	IBM47	M	No	Clinically defined IBM	52	3	non ambulant	No	Severe	Severe	Severe	Severe	17	4	10	3	positive	Y	Y	Y	Y	Y	Y	Y
48	IBM48_1	F	No	Probable IBM	54	1	ambulant unaided	Yes	Mild	Mild	No	No	19	3	9	7	negative	Y	Y	Y	Y	Y	Y	Y
48	IBM48_2	F	No	Probable IBM	55	2	ambulant unaided	Yes	Mild	Mild	No	No	19	3	9	7	negative	Y	Y	Y	Y	Y	Y	Y
48	IBM48_3	F	No	Probable IBM	59	6	ambulant unaided	Yes	Mild	Mild	No	No	19	3	9	7	negative	Y	Y	Y	Y	Y	Y	Y
49	IBM49	F	Yes - hyperCKemia	Clinically defined IBM	73	14	ambulant unaided with difficulties/for short distances	Yes	Moderate	Mild	No	Mild	22	3	12	7	positive	Y	Y	Y	Y	Y	Y	Y
50	IBM50	F	No	Clinically defined IBM	71	4	ambulant unaided with difficulties/for short distances	Yes	Moderate	No	No	Mild	19	3	8	8	positive	Y	Y	Y	Y	Y	Y	Y
51	IBM51	F	No	Clinically defined IBM	80	2	ambulant only with support	No	Severe	No	Mild	Moderate	16	4	8	4	negative	Y	Y	Y	N	Y	Y	Y
52	IBM52	F	No	Clinically defined IBM	64	2	ambulant unaided	Yes	Mild	No	Mild	No	23	2	15	6		Y	Y	Y	N	Y	Y	Y
53	IBM53	M	No	Clinically defined IBM	66	5	ambulant only with support	Yes	Moderate	Mild	Mild	Moderate	20	3	13	4	positive	Y	Y	Y	Y	N	Y	Y
54	IBM54	M	No	Clinically defined IBM	49	1	ambulant unaided	Yes	Mild	No	Mild	Mild	24	3	15	6	positive	Y	Y	Y	N	Y	Y	Y
55	IBM55	M	Yes -Proximal upper limb weakness	Probable IBM	65	8	ambulant unaided with difficulties/for short distances	No	Moderate	Moderate	Moderate	Moderate	19	4	11	4	positive	Y	Y	Y	Y	N	Y	Y
56	IBM56	M	No	Probable IBM	83	1	ambulant only with support	No	Severe	Severe	No	No	24	4	14	6		Y	Y	Y	N	Y	Y	Y
57	IBM57	M	No	Probable IBM	74	9	ambulant unaided with difficulties/for short distances	Yes	Severe	No	No	No	26	3	16	7		Y	Y	Y	Y	N	Y	Y
