# Supplementary material for: Muscle Imaging in Inclusion Body Myositis: Refinement of MRI Criteria and Insights Into Upper Body Involvement
Source: J Cachexia Sarcopenia Muscle. 2026 Jan 19;17(1):e70173. doi: 10.1002/jcsm.70173 (PMC12813550; doi:10.1002/jcsm.70173)
Supplement: Supplementary file 8 — Table S2: Sensitivity of main and supportive criteria across clinical subgroups of IBM patients. [file JCSM-17-e70173-s002.docx]

| **Patients’ subgroups** | **MRI scans fulfilling**  **M1** | **MRI scans fulfilling M2** | **MRI scans fulfilling M3** | **MRI scans fulfilling S1** | **MRI scans fulfilling S2** | **MRI scans fulfilling S3** |
| --- | --- | --- | --- | --- | --- | --- |
| **Disease Classification^a^** | **n.s.^b^** | **n.s.** | **n.s.** | **n.s.** | **n.s.** | **n.s.** |
| Clinicopathologically defined IBM – n. (%) | 40/42  (95%) | 41/42  (98%) | 41/42  (98%) | 29/42  (69%) | 33/42  (79%) | 42/42  (100%) |
| Clinically  defined IBM – n. (%) | 15/15  (100%) | 15/15  (100%) | 15/15  (100%) | 8/15  (53%) | 12/15  (80%) | 15/15  (100%) |
| Probable IBM – n. (%) | 11/11  (100%) | 11/11  (100%) | 11/11  (100%) | 8/11  (73%) | 8/11  (73%) | 11/11  (100%) |
| **Disease Onset** | **p=0.03** | **n.s.** | **n.s.** | **n.s.** | **n.s.** | **n.s.** |
| Typical – n. (%) | 56/56  (100%) | 55/56  (98%) | 56/56  (100%) | 39/56  (70%) | 44/56  (79%) | 56/56  (100%) |
| Atypical – n. (%) | 10/12  (83%) | 12/12  (100%) | 11/12  (92%) | 6/12  (50%) | 9/12  (75%) | 12/12  (100%) |
| **Disease Duration** | **n.s.** | **n.s.** | **n.s.** | **n.s.** | **n.s.** | **n.s.** |
| <5 years – n. (%) | 30/30  (100%) | 30/30  (100%) | 30/30  (100%) | 16/30  (53%) | 24/30  (80%) | 30/30  (100%) |
| 6-10 years – n. (%) | 16/18  (89%) | 18/18  (100%) | 17/18  (94%) | 14/18  (78%) | 13/18  (72%) | 18/18  (100%) |
| >10 years – n. (%) | 20/20  (100%) | 19/20  (95%) | 20/20  (100%) | 15/20  (75%) | 16/20  (80%) | 20/20  (100%) |
| **Swallowing function** | **n.s.** | **n.s.** | **n.s.** | **n.s.** | **n.s.** | **n.s.** |
| Non dysphagic – n. (%) | 35/36  (97%) | 36/36  (100%) | 35/36  (97%) | 25/36  (69%) | 29/36  (81%) | 36/36  (100%) |
| Dysphagic – n. (%) | 31/32  (97%) | 31/32  (97%) | 32/32  (100%) | 20/32  (63%) | 24/32  (75%) | 32/32  (100%) |
| **Walking ability** | **n.s.** | **n.s.** | **n.s.** | **p=0.004** | **n.s.** | **n.s.** |
| Ambulant unaided –n.(%) | 16/17  (94%) | 17/17  (100%) | 16/17  (94%) | 6/17  (35%) | 15/17  (88%) | 17/17  (100%) |
| Ambulant unaided with difficulties/for short distances – n. (%) | 30/31  (97%) | 31/31  (100%) | 31/31  (100%) | 22/31  (71%) | 24/31  (77%) | 31/31  (100%) |
| Ambulant only with support/  non ambulant – n. (%) | 20/20  (100%) | 19/20  (95%) | 20/20  (100%) | 17/20  (85%) | 14/20  (70%) | 20/20  (100%) |
| **Anti-CN1A antibody status** | **n.s.** | **n.s.** | **n.s.** | **n.s.** | **n.s.** | **n.s.** |
| Negative – n. (%) | 12/12  (100%) | 12/12  (100%) | 12/12  (100%) | 8/12  (67%) | 10/12  (83%) | 12/12  (100%) |
| Positive – n. (%) | 18/19  (95%) | 19/19  (100%) | 18/19  (95%) | 14/19  (74%) | 13/19  (68%) | 19/19  (100%) |

^a^ according to the 2011 ENMC research diagnostic criteria [4]; ^b^ n.s.: not significant (p ≥ 0.05); p values are reported only when <0.05.
